# Tired, Worried and Burned Out, but Still Resilient: A Cross-Sectional Study of Mental Health Workers in the UK during the COVID-19 Pandemic

**DOI:** 10.3390/ijerph18094457

**Published:** 2021-04-22

**Authors:** Sofia Pappa, Joshua Barnett, Ines Berges, Nikolaos Sakkas

**Affiliations:** 1 Department of Community Mental Health Services, West London NHS Trust, London UB2 4SD, UK; joshua.barnett@westlondon.nhs.uk (J.B.); ines.berges@westlondon.nhs.uk (I.B.); 2 Division of Brain Sciences, Imperial College London, London SW7 2BX, UK; 3 Department of Adult Community Services, Oxleas NHS Foundation Trust, London SE2 OAS, UK; nicksakkas1@gmail.com

**Keywords:** COVID-19, healthcare workers, United Kingdom, mental health, burnout, resilience, insomnia, depression, anxiety, lifestyle

## Abstract

The burden of the COVID-19 pandemic on health systems and the physical and mental health of healthcare workers (HCWs) has been substantial. This cross-sectional study aims to assess the effects of COVID-19 on the psychological wellbeing of mental health workers who provide care to a vulnerable patient population that have been particularly affected during this crisis. A total of 387 HCWs from across a large urban mental health service completed a self-administered questionnaire consisting of socio-demographic, lifestyle and work-based information and validated psychometric scales. Depression and anxiety were measured using the Patient Health Questionnaire (PHQ-9) and the Generalized Anxiety Disorder Scale (GAD-7), respectively; sleep problems with the Athens Insomnia Scale (AIS); burnout with the Maslach Burnout Inventory (MBI); and resilience with the Resilience Scale-14 (RS-14). Multivariable logistic regression analysis was performed to determine potential mediating factors. Prevalence of burnout was notable, with 52% recording moderate/severe in Emotional Exhaustion, 19.5% moderate/severe in Depersonalisation, and 55.5% low/moderate Personal Accomplishment. Over half of all respondents (52%) experienced sleep problems; the presence of depressive symptoms was a significant predictor of insomnia. An increase in potentially harmful lifestyle changes, such as smoking, alcohol consumption and overeating was also observed. However, high Resilience was reported by 70% of the samples and the importance of this is highlighted. Female gender was associated with increased levels of depression and emotional exhaustion while those with a history of mental health conditions were most at risk of affective symptoms, insomnia, and burnout. Overall, our study revealed considerable levels of psychological distress and maladaptive coping strategies but also resilience and satisfaction with organizational support provided. Findings can inform tailored interventions in order to mitigate vulnerability and prevent long-term psychological sequelae.

## 1. Introduction

The COVID-19 pandemic began with the emergence of a novel coronavirus (SARS-CoV-2) in December 2019, epicentred in Wuhan, China [1]. The first case was confirmed in the UK on 31 January 2020 and by early March, the World Health Organization (WHO) had declared COVID-19 a pandemic [2]. As of April 2021, there has been an estimated 138.8 million global cases of COVID-19, resulting in 2.9 million deaths [3].

The UK has experienced several post-modern pandemics, though their effects have been at large relatively modest. There are estimated to be approximately 105,000 people in the UK living with human immunodeficiency virus/acquired immune deficiency syndrome (HIV/AIDS), which represents a small percentage of the total population [4,5] and the 2003 SARS epidemic did not cause the devastating health impact that many feared [6]. In addition, the Influenza A (H1N1) epidemic resulted in significantly fewer fatalities compared to COVID-19 [7] and did not cause such disruption to the way of life or the healthcare system. However, a survey of healthcare workers (HCWs) during the H1N1 outbreak did reveal moderately high levels of anxiety in over half of those who participated [8].

An early position paper [9] called for high-quality research into the mental health effects of the COVID-19 pandemic across the population and on high risk groups, like HCWs, so that effective interventions can be established. Following the application of a national lockdown, approximately six weeks after the first UK COVID-19 case, increased levels of mental distress [10], depression, and anxiety [11] were described in studies that were conducted on the general population. Women, young people, the socially disadvantaged, and those with a pre-existing mental health condition were reported to be at risk of worse mental health outcomes across most factors [12]. Another community cohort study also showed that women and young people were at greatest risk of psychological morbidity [11].

Furthermore, early studies and rapid systematic reviews demonstrated that HCWs in Asia have been susceptible to various mental health concerns during the outbreak including fear, anxiety, depression, insomnia, traumatic stress, and burnout [13,14,15]. Subsequent studies confirmed a similar picture emerging from other hot spots of the outbreak in Europe, including the UK [16,17,18]. Lack of personal protective equipment (PPE) and routine testing, staff shortages, inadequate training prior to redeployment, rapidly changing guidelines, risk of transmission of infection to friends and family, media reports of HCWs becoming unwell, and physical exhaustion are just some of the contributory factors [19,20,21]. HCWs working in mental health may be faced with additional challenges as they provide care to a highly vulnerable patient group. Furthermore, the role of burnout [22,23] and the importance of resilience [24] amongst National Health Service (NHS) staff had already garnered increased interest in recent years; in 2017, The Lancet went as far as stating that physician burnout had reached “epidemic levels” [25]. 

The aim of this study was to examine the effects of COVID-19 outbreak on the wellbeing, sleep and lifestyle changes alongside the levels of burnout and resilience on healthcare professionals working in a mental health setting. Ideally, the results can help, in conjunction with existing evidence, to inform on interventions that may protect and improve the mental health of staff during the ongoing, as well as future, epidemics.

## 2. Materials and Methods

### 2.1. Study Design and Population

We conducted a cross-sectional study in a large urban mental health trust in West London, comprising of community and inpatient services (including dedicated COVID-19 wards) from the beginning of June to the end of July 2020. All staff were invited to participate in this self-administered, anonymous, and confidential online survey. The participants were self-selected and were allowed to terminate the survey at any point if they wanted to. The study was approved by the department for audit and naturalistic research (project number 1809).

### 2.2. Questionnaire 

The self-reported questionnaire was divided into three sections and captured the following data: socio-demographic information, medical and psychiatric history, work environment, lifestyle, as well as psychometric scales assessing levels of burnout, resilience, anxiety, depression, insomnia, and fear:

- Socio-demographic: age, gender, ethnicity, home living situation, occupation, area of occupation (community, forensic, inpatient, COVID-19 ward etc.).
- Personal and work-related effects of COVID-19: Determining if participants are “high risk”, if they/members of their household had contracted the virus, if they have had to self-isolate or “shield”, how much face-to-face contact they have with patients (none, occasional, regular). How well supported and informed they are at work, worries about becoming infected or infecting others with COVID-19 and the consequences of this—these items were rated on a 5 = point Likert type scale; participants were asked how much they agreed with the statements presented, with answers ranging from strongly agree to strongly disagree.
- Mental health and lifestyle-related effects of COVID-19: Pre-existing mental health diagnoses, changes to lifestyle (alcohol/tobacco/drug use, exercise), as well as psychological impact (perceived stress, sleep, nightmares, self-harm/suicidal thoughts), awareness and access of wellbeing support within the organization, and psychometric scales as detailed below.

### 2.3. Psychometric Scales

- Burnout: Maslach Burnout Inventory (MBI) is a 22-item questionnaire which assesses three dimensions: emotional exhaustion (EE, 9 items), depersonalization (DP, 5 items), and personal accomplishment (PA, 8 items) [26]. Higher scores in the EE and DP dimensions indicate more severe burnout, whereas higher scores in the PA subscale indicate less burnout. Cut-offs for moderate and severe EE were ≥17 and ≥27, for moderate and severe DP ≥ 7 and ≥ 13, and for moderate and severe reduced PA ≤ 38 and ≤21.
- Resilience Scale-14 (RS-14) is a modified, consistent, and validated version of the RS-25 questionnaire [27], consisting of 14 self-reported items which are measured on a 7-point Likert-type rating scale ranging from 1 (strongly disagree) to 7 (strongly agree). Scores range from 14 to 98 in total; <65 indicate “low resilience”, 65–81 “moderate resilience” and >81 “high levels of resilience” [28].
- Patient Health Questionnaire-9 (PHQ-9) is a nine-item self-administered screening tool for depression [29]. The scale investigates symptom severity over the past two weeks. Items are rated on a 4-point Likert type scale, ranging from 0 (not at all) to 3 (nearly every day). Total scores range between 0 and 27; scores of 0–4 are regarded as “minimal or none,” 5–9 as “mild,” 10–14 as “moderate,” 15–19 as “moderately severe,” and 20–27 as “severe”. The recognized cut-off point of 10 or greater corresponds to moderate to severe symptomatology indicative of a clinically significant problem.
- General Anxiety Disorder-7 (GAD-7) is a seven-item self-reported anxiety scale evaluating symptom severity in the preceding two weeks [30]. Items are rated on a 4-point Likert-type scale, ranging from 0 (not at all) to 3 (nearly every day). Total scores range between 0 and 21. Total scores of 0–4 were regarded as “not at all,” 5–9 as “mildly,” 10–14 as “moderately,” and 15 as “severely”. A cut-off point of 10 or greater is commonly used for case definition.
- Athens Insomnia Scale (AIS) is an eight-item self-reported questionnaire designed for quantifying sleep difficulty based on the ICD-10 criteria over the last month, which has shown good consistency, reliability, and validity [31]. The items are rated on a 4-point Likert-type scale, ranging from 0 (no problem or equivalent meaning) to 3 (severe problem or equivalent meaning). The commonly accepted cut off score is 6, with higher scores indicating more severe insomnia [32].
- A numerical fear rating scale (NFRS) was used to measure the level of fear in the study which has been reported to have good reliability and validity [33]. It is a segmented numeric version of the visual analogue scale (VAS) in which a respondent selects a whole number (0–10 integers) that best reflects the intensity of their fear. Higher scores indicate greater fear as follows: 0 for no fear, 1–3 for mild fear, 4–6 for moderate fear, 7–9 for severe fear, 10 for extreme fear.

### 2.4. Statistical Analysis

Statistical analysis was performed using SPSS v25 (IBM, Armonk, NY, USA). Descriptive statistics were used to present socio-demographics and other COVID-related information and continuous outcome variables including, fear, anxiety, depression, traumatic stress, and burnout; categorical variables were expressed as absolute values (percentages) and continuous variables as mean values ± (standard deviation). Welch’s t-test and Multivariate Analysis of Variance (MANOVA) were used to examine the association between continuous variables. Multivariable logistic regression was used to determine independent associations of binary outcomes. Two-tailed *p* values of less than 0.05 were deemed statistically significant.

## 3. Results

### 3.1. Participant Characteristics

In total, 387 responses were recorded. The sample was principally white (67.8%), female (71.1%), aged 51–65 (31.1%), who live with a partner (48%) and work in community services (32.2%). Sociodemographic and basic work-related information is displayed in Table 1.

**Figure 1:**
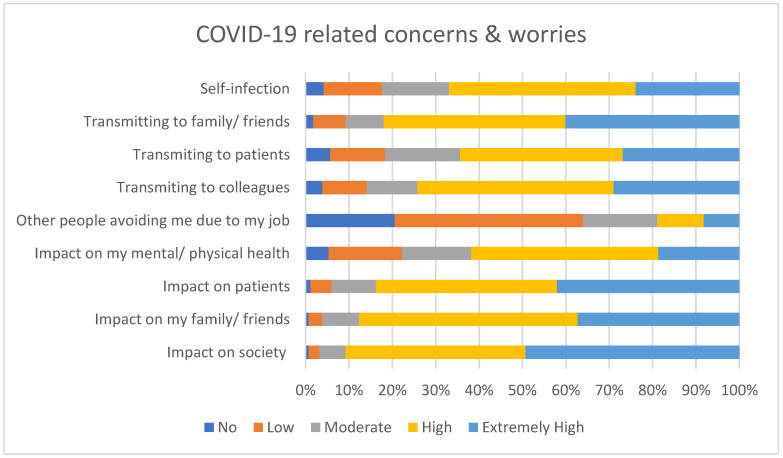
Self-reported concerns about COVID-19.

**Figure 2:**
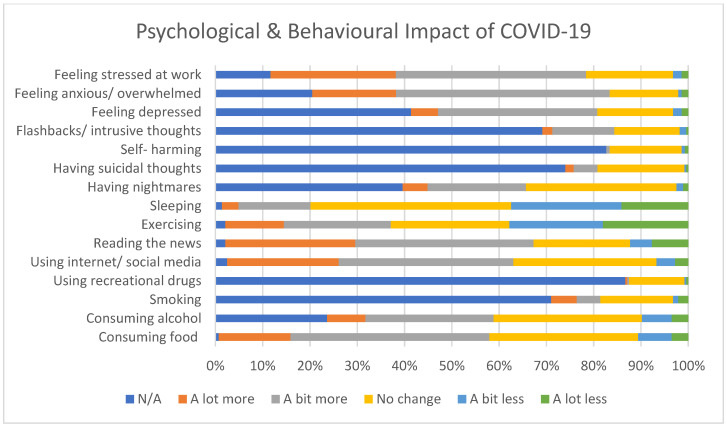
Self-reported behavioural and lifestyle impact of COVID-19.

The majority of respondents were concerned about self-infection, transmitting infection to friends, family, patients, and colleagues and the impact COVID-19 would have on patients, friends, family, and society as a whole, as seen in Figure 1. Figure 2 displays the self-reported psychological and behavioural impact of COVID-19, with over half of the participants reporting feeling more stressed and overwhelmed at work. A small proportion also reported an increase in suicidal thoughts and smoking, with much larger percentages consuming more food and alcohol.

**Figure 3:**
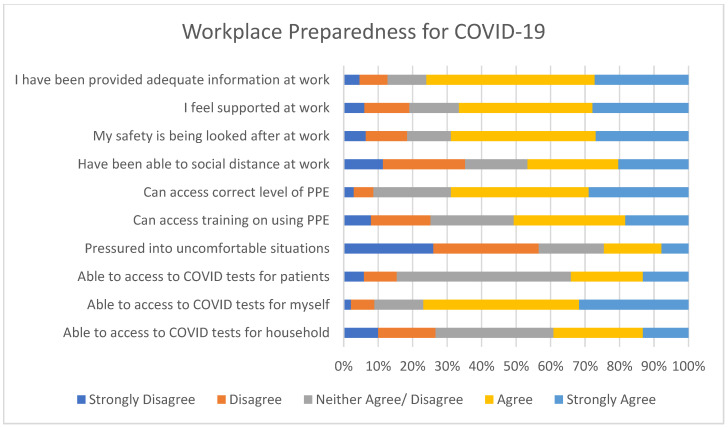
Self-reported workplace preparedness for COVID-19.

As Figure 3 shows, a large majority felt as though they had been provided with adequate information at work, that they were well supported, that their safety was being suitably looked after, and they had sufficient access to PPE.

### 3.2. Psychometric Scales Outcomes

**Figure 4:**
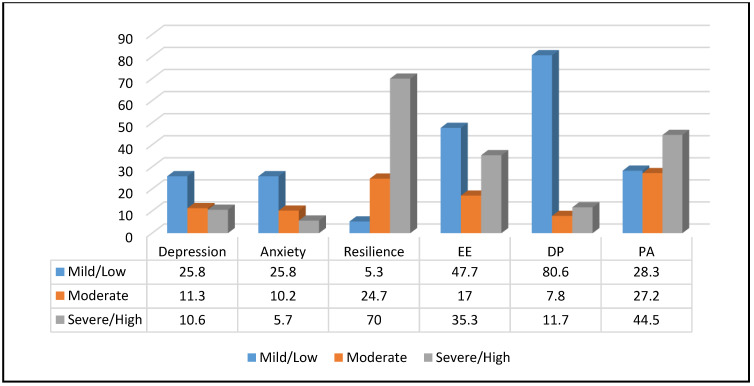
Severity of Depression, Anxiety, Burnout, and Resilience.

A substantial amount of HCWs reported at least mild depression, anxiety, and/or burnout. Insomnia was seen in over half of all respondents, while high levels of resilience were seen in a large majority (Table 2 & Figure 4). Table 3 indicates that females demonstrated significantly higher levels of anxiety and EE compared to males and those with a pre-existing mental health condition recorded higher mean scores in depression, anxiety, insomnia, and EE; they also were noted to score lower in resilience.

The proportion with moderate/severe depression was 21.9%, whilst moderate/severity anxiety was 15.9%. Lower resilience (OR: 0.79, *p* < 0.001), alcohol consumption (OR: 1.83, *p* = 0.014), feeling pressured to work in uncomfortable situation (OR: 2.42, *p* = 0.026), self-harming (OR: 0.39, *p* = 0.038) and insomnia (OR: 0.01, *p* < 0.001) were significantly associated with a higher likelihood of exhibiting symptoms of depression. The presence of depressive symptoms (OR: 1.98, *p* < 0.001), concerns about COVID impacting own mental and physical health (OR: 0.07, *p* = 0.012), concerns about transmitting COVID to patients (OR: 0.28, *p* = 0.014) and to colleagues (OR: 3.77, *p* = 0.017), access to PPE (OR: 3.48, *p* = 0.037) and training on using PPE (OR: 2.96, *p* = 0.042) predicted anxiety. 51.6% of participants experienced sleep problems; the only statistically significant predictor of insomnia noted from logistic regression was the presence of depressive symptoms (OR: 0.66, *p* < 0.001).

Elevated levels of burnout were reported in all three domains: 17% experienced moderate and 35.3% high EE. Moderate DP was seen in 7.8% and this was high in 11.7%. PA was low in 28.3%, moderate in 27.2%, and high in 44.5%. The multinomial logistic regression analysis model revealed that insomnia (OR: 0.26, *p* = 0.014), perceived stress (OR: 3.49, *p* < 0.001), concerns about the impact of COVID on society (OR: 0.43, *p* = 0.036), concerns about transmitting COVID to family/ friends (OR: 0.42, *p* = 0.03) or patients (OR: 2.18, *p* = 0.045), access to COVID tests for family (OR: 0.57, *p* = 0.038), and being pressured at work (OR: 1.83, *p* = 0.016) were all significant predictors of EE. Fear of COVID (OR: 0.5, *p* = 0.003), self-harm (OR: 0.42, *p* = 0.038), concerns about the impact of COVID on society (OR: 0.26, *p* = 0.041), being pressured at work (OR: 2, *p* = 0.048), availability of training on PPE use (OR: 1.95, *p* = 0.035), whether one is feeling supported at work (OR: 0.15, *p* = 0.003) and receiving appropriate information at work (OR: 3.28, *p* = 0.011), work stress (OR: 2.98, *p* = 0.026), and exercising (OR: 0.5, *p* = 0.014) were significantly associated with DP. Resilience (OR: 0.88, *p* < 0.001), fear of COVID (OR: 1.4, *p* = 0.003), concerns about the impact of COVID on patients (OR: 2.03, *p* = 0.037), concerns about transmitting COVID to patients (OR: 2.51, *p* = 0.024), and family/ friends (OR: 0.41, *p* = 0.013) receiving appropriate information at work (OR: 1.95, *p* = 0.047) and concerns about self-contamination (OR: 2.01, *p* = 0.014) were found to correlate with a lower sense of PA. 

Furthermore, 70% of respondents scored high in resiliency, 24.7% moderate and 5.3% low. Concerns about transmitting COVID to patients (OR: 0.28, *p* = 0.05) and whether one feels supported at work (OR: 0.29, *p* = 0.39) were significant predictors of resilience. 

## 4. Discussion

To our knowledge, this is the first study in the UK to report on the impact of COVID-19 on the mental health and lifestyle habits of HCWs solely within a mental health setting. The results show considerable levels of burnout, insomnia, depression, and anxiety, as well as an increase in potentially harmful behaviours such as smoking, alcohol consumption, and overeating. Furthermore, the study identifies a number of potential predictive or mediating factors and displays the levels of resilience amongst the respondents, remarking upon its importance at this trying time for the global healthcare community.

Previous studies have shown higher rates of depression and burnout amongst psychiatrists compared to other medical specialties [34,35], which have been attributed to both personal and organisational factors [36] and may well apply across other healthcare professionals in mental health. Hence, a targeted study, such as this one, may allow for future comparisons within and between multi-disciplinary teams across different settings and specialties. Nevertheless, prevalence rates in our sample were generally comparable to psychological outcomes previously reported among HCWs in other countries, such as China and Singapore [14], Italy [37], and Greece [38]. Though, some of these may have experienced different transmission rates and pressures on healthcare services at the time. In addition, comparisons between studies should be carried out mindfully, given that the use of alternative rating scales and cut offs promotes inherent heterogeneity [36]. 

This study adds to the growing body of evidence showing the potential psychological cost of the outbreak on HCWs. It highlights the necessity of tailored interventions in order to support wellbeing and prevent the onset of enduring psychological sequelae; this is all-important at this time given the crucial role they are performing more than a year into the pandemic.

### 4.1. Mood and Sleep

One in five HCWs experienced at least mild depressive symptoms and one in five moderate to severe. A similar proportion reported some degree of anxiety, with mild symptoms present in 26% and moderate to severe in 16%. The largely similar prevalence of anxiety and depression is comparable to the one reported in an early rapid systematic review with meta-analysis of 13 studies from Asia (23.2% and 22.8% respectively) [14]. A subsequent meta-analysis [13] found that rates of depression were highest among those with a pre-existing mental health condition; this was also noted in our survey alongside higher levels of anxiety and insomnia. 

Rates of moderate/severe depression and anxiety amongst HCWs were similar to those recorded in the general population at roughly the same period of time (22.1% & 21.6% respectively) [39]. Logistic regression showed that higher levels of anxiety, lower levels of resilience, increased alcohol consumption, and increased self-harming were significant predictors of depression. Concerns about transmitting COVID-19 to patients and colleagues and lack of access to PPE were associated with development of anxiety symptoms. A community study from April 2020 showed that female sex and younger age were both predictors for higher levels of depression, anxiety, and stress [11].

Over half (52%) of participants recorded scores indicative of sleep problems and/or insomnia. This figure is considerably higher than the one quoted in the above meta-analysis (38.9%) as well as from a separate systematic review of 59 studies worldwide (37%) [40]. Nevertheless, it is still lower than the reported levels of sleep disturbance amongst paediatric HCWs across Italy for example (67.4%), which was one of Europe’s hardest hit areas [41]. To our knowledge, there are no studies available for comparison that used validated rating scales to investigate the prevalence of insomnia in the general UK population during this period of time.

In our study the strongest predictor for insomnia was the presence of depressive symptoms (OR: 0.66, *p* < 0.001). A study by Wang et al. [42] also identified concurrent depression as a statistically significant risk factor, but noted that being exposed to COVID-19 patients was more strongly correlated. Another study, from multiple centres in China, showed that exposure to COVID-19 increased the likelihood of developing insomnia [43]. It is possible that this relationship was not established within our study given the small sample size of respondents who worked on dedicated COVID-19 wards at the time.

### 4.2. Lifestyle Changes

A considerable proportion of participants responded that they were smoking, drinking, and eating more and exercising less during the pandemic. Of those who smoked in our survey (29% of all respondents), 36% reported smoking more than they were pre-pandemic. Stress and worsening mental health are both predisposing factors for increased smoking [44] and recent qualitative data from the UK postulate that smoking was being used as a coping mechanism to deal with anxiety, boredom, stress, and anger during lockdown [45]. Alcohol intake appeared also on the rise, with similar rates recorded in our study and a recent community survey (35.3% and 36%) [46]. Likewise, a separate study demonstrated an increase of binge-drinking from 10.8% to 16.2% in the general population [47] In fact, the effects of the increased use of alcohol and cigarettes may be a “hidden” consequence of this outbreak that may only become noticeable in the long run.

Further behavioural changes are noteworthy within the study population. For example, 38% reported exercising less. A similar proportion of people were found to be exercising less in a recent survey of the general population (40%) [46]. The reasons for decreased levels of exercise is likely multifactorial, for example application of lockdown restrictions and closure of gyms. Regular exercise is protective against developing depression, while physical inactivity is a risk factor for affective symptoms [48] and whilst no link was seen between depressive symptoms and reduced exercise in our study, the long-term effects may be different. 

In the survey by Robinson et al., 42% of respondents also reported that they had “eaten more because of my feelings” and 36% felt they were not in control of their eating. Whilst 10% of those from our survey were eating less, a majority (57%) were consuming more food. This could well be attributed to “comfort eating”, i.e. eating induced by negative affect [49] as also supported by a large population based study [50] whereby individuals with depressive symptoms were more likely to report a change in eating habits.

### 4.3. Burnout

High levels of burnout were reported by survey respondents: more than half showed moderate to high EE and low to moderate PA and one in five demonstrated moderate to high DP. The regression model showed that having concerns about the impact of Covid on society and feeling pressured or uncomfortable at work were both predictors for higher levels of EE and DP, whereas concerns about transmitting COVID-19 to family, friends, and patients were associated with increased levels of EE and a lower sense of PA.

In 2019, the WHO added Burnout to the 11th Revision of the International Classification of Diseases (ICD-11) and defined it as “an occupational phenomenon… resulting from chronic workplace stress that has not been successfully managed”. However, burnout is not a new concept and a systematic review from 2017 [51] examining literature from the preceding 20 years reported on the presence of moderate to high EE (range 31–54.3%) and DP (17.4–44.5%) as well as low PA (6–39.6%) in doctors in the UK. Presently, there is limited research into the levels of burnout amongst HCWs during pandemic in the UK, though, in a recent BMA survey, 51% of doctors self-reported experiencing at least one of the following: depression, anxiety, stress, or burnout [52] but validated rating scales were not employed. Similar burnout levels were also recorded in other areas such as Italy—one of the harder hit regions during the initial stages of the outbreak (EE 67%, DP 26% and low PA 60%) [53]. Interestingly, a multi-centre study from Greece [38] reported even greater levels of burnout (EE 69%, DP 86% and low PA 50%) despite the very benign course of the outbreak during the same period of time. The variation in reported figures is likely to be multifactorial, but differences in infrastructure and socioeconomic disparity could account for this. 

A common view within the NHS is that it often “runs on goodwill” [54,55,56], relying on staff regularly working extra hours and “going the extra mile” to keep the system in a state of flow. Excessive levels of burnout may have a significant impact on the system’s ability to function and cope under the current circumstances, given the known associations between burnout and reduced productivity, increased physician turnover, and absenteeism as well as medical errors, accidents, and suicidality [57]. In fact, of the staff we surveyed that experience suicidal thoughts (25.9% of all respondents), 26.2% reported an increase in suicidal ideation. Given reports of suicide amongst HCWs as a result of the pandemic alongside the already higher risk of suicide compared to the general population [58], this figure may be concerning.

The importance of counteracting the rising tide of burnout is clear; and organisational, structural, and individual interventions have been called for both prior to [25,59] and since the onset of [60] the pandemic. Worries about self-contamination and spread of infection to friends, family, and patients, which all contributed to burnout, have possibly been lessened with the largely successful deployment of the vaccination program. Better provision of PPE could also help in this regard, especially given that concerns about its availability were noted to further the prevalence of burnout in both this survey and others [61]. On a personal level, meditation has been mooted as an easily deliverable and effective intervention [62]. Successful application of this could pave the way for other interventions such as mindfulness and small group peer support. Furthermore, 86.6% of those surveyed were aware that psychological support was available for staff; yet, only 12.5% made use of it. A better understanding into why this is the case would be useful in improving uptake.

### 4.4. Resilience

A large majority (70%) of respondents reported high levels and a further 25% moderate levels of resilience. Previous studies suggest that being female [63,64] and feeling supported at work [65] inferred higher levels of resilience. Whilst there was no statistical difference between genders in this study, better support was indeed found to be a significant predictor.

Since its inception in 1948, NHS staff have not been exposed to this many mortalities over such a short period of time and resilience is viewed as a key trait for coping through the continuous exposure to death, distress, and moral injury [65]. Furthermore, it is an important quality for effective clinical leadership [66] and its proliferation may even help to reverse the trend of medical staff leaving their profession [67]. As with burnout, interventions could be put into practice that improve clinical processes and reduce clerical burden on clinicians in order to enhance resilience [68]. An article in the BMJ from 2015 [65] argued that fostering a supportive and positive culture will create a more resilient environment, though an alternative view is that resilience is more dynamic and can be developed or improved upon [69]. Suggestions include mindfulness [70] and resilience workshops [71]. 

### 4.5. Strengths & Limitations

Demographically, the study shows good variation in the domains of age groups, professions, areas of work, and ethnicity (though, those of a black, Asian and minority ethnic BAME background are somewhat overrepresented whilst those of any white background underrepresented when compared to NHS statistics) [72]. Females appear disproportionately represented within the study (71.1%), but this is largely in keeping with gender representation within the NHS as a whole [73]. Additionally, there is evidence to suggest that men are less likely to respond to health-related surveys in general when compared to women [74] and that they have a tendency to underreport depressive symptoms [75]. The cross-sectional nature of the study does not allow for the inference of causality, which is a key limitation. Additionally, being an online questionnaire means that self-selection bias, coupled with a relatively small sample size, limits the generalisability of the findings.

Mental health symptoms were self-reported rather than professionally assessed, meaning a clinical syndrome may not be present even if a “cut off” score is surpassed. However, the purpose of this study was to screen for potentially concerning symptoms rather than to diagnose mental illness. A strength of this study was establishing the presence or absence of a pre-existing mental health condition; failure to do this has been cited as a limitation in multiple other similar studies [10,52]. Finally, this study is only a snapshot of the pandemic and longitudinal studies would be required to assess longer term mental health outcomes.

## 5. Conclusions

Perhaps unsurprisingly, a high prevalence of burnout and, most commonly, insomnia was seen amongst staff within a large mental health trust during the current COVID-19 outbreak. An increase in potentially damaging lifestyle changes were also noted. At the same time, the majority of participants reported high levels of resilience and a good level of organisational support. Study findings can be utilised to inform upon targeted interventions that can help address areas of concern during this global health crisis.

## Figures and Tables

**Table 1:** Sample Characteristics.

Characteristics	*N*	%
Age		
16–20	1	0.4
21–30	64	22.6
31–40	57	20.1
41–50	71	25.1
51–65	83	29.3
66+	7	2.5
**Gender**		
Male	78	26.6
Female	201	71
Prefer not to say	4	1.4
**Race and Ethnicity**		
All White backgrounds	189	66.8
All Black backgrounds	27	9.5
All Asian backgrounds	41	14.5
Mixed backgrounds	11	3.9
Other	15	5.3
**Occupation**		
Doctors	45	16
Nurses	46	16.4
Psychologists	52	18.5
Health Care Assistant	21	7.5
Administrative/ Management	41	14.6
Other	76	27
**High-risk group for COVID-19**		
Yes	68	24
No	215	76
**Current Contact with Patients**		
No	104	39.2
Yes, occasionally	72	27.2
Yes, regularly	89	33.6
**Pre-existing Mental Health Condition**		
Yes	60	78.8
No	223	21.2
**Adequacy of support offered at work**		
Yes	104	37.3
Yes, to some extent	115	41.2
No	60	21.5

**Table 2:** Prevalence and Severity of Insomnia, Depression, Anxiety, Burnout, and Resilience.

Insomnia		Emotional Exhaustion	
No Insomnia	48.4%	Low	47.7%
Insomnia	51.6%	Moderate	17%
		High	35.3%
**Depression**		**Depersonalisation**	
Mild	25.8%	Low	80.6%
Moderate	11.3%	Moderate	7.8%
Severe	10.6%	High	11.7%
**Anxiety**		**Personal Accomplishment**	
Mild	25.8%	Low	28.3%
Moderate	10.2%	Moderate	27.2%
Severe	5.7%	High	44.5%
**Resilience**			
Low		5.3%	
Moderate		24.7%	
High		70%	

**Table 3:** Psychometric Scales: Mean scores by sex and presence/absence of pre-existing mental health conditions.

Domain	Mean ± Std. Error			df	*P*-Value	Cohen’s *d*
	Male	Female	*t*			
***PHQ-9***	4.72 ± 0.70	6.31 ± 0.40	1.98	129.68	0.05 *	0.27
***GAD-7***	3.82 ± 0.57	5.06 ± 0.34	1.87	135.50	0.064	0.25
***AIS***	5.95 ± 0.63	7.11 ± 0.40	1.57	139.08	0.119	0.21
***MBI_EE***	18.35 ±1.47	22.38 ± 0.94	2.32	143.66	0.022 *	0.31
***MBI_PA***	31.15 ± 1.29	32.81 ± 0.70	1.13	124.66	0.262	0.16
***MBI_DE***	4.32 ± 0.54	3.61 ± 0.31	−1.14	130.77	0.709	0.15
***RS_14***	83.05 ± 1.34	83.65 ± 0.72	0.40	123.26	0.694	0.05
	**No Pre-existing MH diagnosis**	**Pre-existing MH diagnosis**	***t***	**df**	***p*-Value**	**Cohen’s *d***
***PHQ-9***	4.75 ± 0.33	5.01 ± 0.39	−5.38	75.81	<0.001 *	0.85
***GAD-7***	3.81 ± 0.28	8.08 ± 0.74	−5.38	77.10	<0.001 *	0.85
***AIS***	5.98 ± 0.34	9.72 ± 0.78	−4.40	83.03	<0.001 *	0.67
***MBI_EE***	20.12 ± 0.89	24.98 ± 1.67	−2.57	95.18	0.012 *	0.37
***MBI_PA***	32.01 ± 0.71	33.83 ± 1.19	−1.31	104.54	0.192	0.18
***MBI_DE***	3.53 ± 0.29	4.68 ± 0.62	−1.69	87.75	0.094	0.25
***RS_14***	84.44 ± 0.68	80.07 ± 1.54	2.60	83.20	0.011 *	0.39

PHQ-9 = Patient Health Questionnaire-9; GAD-7 = General Anxiety Disorder-7; AIS = Athens Insomnia Scale; MBI_EE = Maslach Burnout Inventory, Emotional Exhaustion; MBI_PA = Maslach Burnout Inventory, Personal Accomplishment; MBI_DE = Maslach Burnout Inventory, Depersonalization; RES-14 = Resilience Scale-14; * = Statistically Significant.

## Data Availability

The data presented in this study are available on request from the corresponding author.
